# The assessment of developmental status using the Ages and Stages questionnaire-3 in nutritional research in north Indian young children

**DOI:** 10.1186/1475-2891-12-50

**Published:** 2013-04-23

**Authors:** Ingrid Kvestad, Sunita Taneja, Tivendra Kumar, Nita Bhandari, Tor A Strand, Mari Hysing

**Affiliations:** 1 Department of Biological and Medical Psychology, Faculty of Psychology, University of Bergen, Bergen, Norway; 2 Regional Centre for Child and Youth Mental Health and Child Welfare, West, UniHealth, UniResearch, Bergen, Norway; 3 Society for Applied Studies, New Delhi, India; 4 Society for Essential Health Action and Training, New Delhi, India; 5 Centre for International Health, University of Bergen, Bergen, Norway; 6 Innlandet Hospital Trust, Anders Sandvigsgate 17, Lillehammer, 2629, Norway

**Keywords:** Developmental assessment, Ages and Stages questionnaire, India, Global health, Health survey, Nutritional epidemiology

## Abstract

**Objective and background:**

For large epidemiological studies in low and middle-income countries, inexpensive and easily administered developmental assessment tools are called for. This report evaluates the feasibility of the assessment tool Ages and Stages Questionnaire 3.edition (ASQ-3) “home procedure” in a field trial in 422 North Indian young children.

**Methods:**

ASQ-3 was translated and adjusted for a North Indian Hindi setting. Three examiners were trained by a clinical psychologist to perform the assessments. During the main study, ten % of the assessments were done by two examiners to estimate inter-observer agreement. During all sessions, the examiners recorded whether the scoring was based on observation of the skill during the session, or on caregiver’s report of the child’s skill. Intra class correlation coefficient was calculated to estimate the agreement between the raters and between the raters and a gold standard. Pearson product moment correlation coefficient and standardized alphas were calculated to measure internal consistency.

**Principal findings:**

Inter-observer agreement was strong both during training exercises and during the main study. In the Motor subscales and the Problem Solving subscale most items could be observed during the session. The standardized alphas for the total ASQ-3 scale across all ages were strong, while the alpha values for the different subscales and age levels varied. The correlations between the total score and the subscale scores were consistently strong, while the correlations between subscale scores were moderate.

**Conclusions/significance:**

We found that the translated and adjusted ASQ-3 “home procedure” was a feasible procedure for the collection of reliable data on the developmental status in infants and young children. Examiners were effectively trained over a short period of time, and the total ASQ scores showed adequate variability. However, further adjustments are needed to obtain satisfying alpha values in all subscales, and to ensure variability in all items when transferred to a North Indian cultural context.

## Background

Poverty, poor health, poor nutrition and deficient care are major risk factors for brain development and cognitive functioning in infants and young children in low and middle-income countries. Large epidemiological studies are needed to identify modifiable risk factors and to clarify their relative importance for development [1,2]. For this purpose, easily administered and inexpensive assessment tools ensuring reliable and valid measurements of developmental status are called for.

There is a range of quality criteria recommended for the assessment of child development in low and middle-income countries. In their toolkit prepared for the World Bank Human Developmental Group, Fernald, Kariger, Engle and Raikes [3] emphasize the significance of careful cultural adjustments of tools, and of assessing the psychometric qualities of the tools within the given cultural context [3]. Further, they recommend inexpensive items and materials that are easily obtainable, easy to use and enjoyable for the child. The assessment tool should meet the total range of the assessed children in order to discriminate between groups of children. Assessment tools that can be used in a wide age range and that are easily adaptable to a given cultural context are recommended.

There are official recommendations for standards in the translational and adaptation process when transferring an instrument to a given cultural setting [4]. Furthermore, unique cultural beliefs, expectations and norms in child-rearing practices may give rise to cultural specific variations in how children develop their skills. Therefore, when an instrument is transferred to a new cultural setting, its original standardized norms or population-based cut off scores are of little value. With no determined norms or cut offs, the utility of a tool should be limited to the comparison between groups, and not as a tool to diagnose or determine developmental delays [3].

Comprehensive tests of development such as the Bayley Scale of Infant and Toddler Development and the Mullen Scales of Early Learning are often referred to as the gold standard in the developmental assessment of infants and young children [5]. These tests offer comprehensive information on a child’s current developmental status, are administered directly with the child, offer normative scores for the areas which are assessed, and psychometric qualities are shown to be highly satisfactory [6]. The comprehensive development tests require thorough training of personnel, preferable professionals with clinical experience within the age group, and administration is often time-consuming. Normative data are commonly limited to the country where the comprehensive tests are created and the process of obtaining normative data for other populations is time-consuming.

The briefer approaches to developmental assessments are often developed as screening instruments designed to identify children with developmental delays who require further assessment [7]. Common factors of screening instruments are that they are efficient to administer and have standardized population-based cut off scores [8]. Screening instruments will differ however, in comprehensibility, i.e. the assessment of simple milestones or of wider aspects of children’s development, in whether they are based on direct assessment with the child or on secondary reports from informants close to the child, and in terms of psychometric qualities [3,8].

The Ages and Stages Questionnaire 3 ed. (ASQ-3) is a widely used screening tool for infants and young children’s development assessing development in five domains: Communication, Gross Motor, Fine Motor, Problem Solving and Personal Social [9]. The screening tool has been developed in the US, has a US standardized sample and its use has been widespread in assessing development in children aged 1 – 66 months at a low cost with cut off scores identifying developmental delays. The ASQ-system is originally a parent-completed questionnaire, but it may also be completed by a professional interacting with the child and caregiver referred to as the “home procedure” in the ASQ-3 manual. Psychometric parameters of the original ASQ-3 have been examined based on completion of a normative sample of approximately 18 000 respondents [9].

The ASQ-3 is available in English and Spanish versions. However, earlier versions of the ASQ have been translated and validated into several languages, including French, Turkish, Korean, Norwegian and Dutch [8,10-13]. There is one study evaluating the ASQ-2 for clinical purpose in four age groups in North India. Reports from the study showed correlations from 0.76 to 0.80 between scores on ASQ and DASII, a comprehensive developmental test widely used in India. In addition, the psychometric properties were reported to be good although with some variations across age groups. Based on their results, the report concludes that the ASQ had satisfactory usability for developmental screening in a North Indian setting [14]. There are also reports of the use of ASQ-2 for research purpose. For example in a multinational study (“Magpie Trial”) involving several countries across different continents [15], a Norwegian randomized placebo-controlled intervention trial in very low birth weight infants [16], and a study in vulnerable children in the Andean region, Ecuador [17].

Reports from studies involving the ASQ-2 commonly describe that changes to items in the ASQ forms were necessary to ensure validity in the given cultural context [10,11,13,17]. In the Indian ASQ study for example, questions concerning mirrors and forks were mostly left unanswered, most likely because of its cultural inappropriateness. Furthermore, many developmental assessment tools, including ASQ-3, are dependent upon parent-completion. Hence, a challenge when collecting data on the developmental status of children in low and middle-income countries is the caregiver’s possible illiteracy and/or inexperience with questionnaires. To get past this challenge, it is widespread in research to use comprehensive tests assessing the child directly, although these tools may be both time and resource consuming. The ASQ-3 “home procedure” where lay-people can be trained to perform the assessment on the child directly may represent an efficient alternative approach in both time and resources for the collection of data on child development in larger studies. The ASQ-2 “home procedure” was used in the previously mentioned study in Ecuador due to caregiver’s inability to complete the questionnaires [17].

Studies have shown that the ASQ-2 is promising as a developmental assessment in low and middle-income countries although cultural adjustments are necessary. However, to our knowledge, little has been done to evaluate the ASQ-3 “home procedure” in epidemiological studies. Hence, how to effectively train multiple examiners and conduct the assessment in large studies ensuring reliable data is not known. The overall aim of the current report is to assess the feasibility of the ASQ-3 “home procedure” for measuring developmental status in young children in a field trail in New Delhi, India. The report addresses necessary alterations and translations in the forms when transferring the ASQ-3 to a North Indian setting, as well as cultural adjustments in the administration of the home procedure. We assessed whether it was possible to, over a short time period, effectively train examiners to collect reliable data on infants and young children’s developmental status by the ASQ-3 “home procedure”, and we measured the internal consistency of the ASQ-3 when transferred to a North Indian Hindi version.

## Methods

### Ethic statement

The study was approved by the ethics committees of the Society for Essential Health Action and Training (India), Society for Applied Studies (India), Christian Medical College (India), and the Norwegian Regional Committee for Medical and Health Research Ethics (REK VEST).

### Participants

The sample was part of a randomized, doubled blind, placebo controlled trial to measure the efficacy of routine administration of folic acid and vitamin B12 to prevent childhood infections (clinicaltrials.gov: NCT 00717730). One thousand children were enrolled and we included the last 440 enrolled children for developmental assessment. The study site was in the low and middle socioeconomic settings of Tigri and Dakshinpuri in New Delhi. The areas are typical urban neighbourhoods with a total population of about 300,000.

For the main study, a door-to-door survey was conducted to identify households with children aged six to 30 months. Children were then screened for eligibility by a physician and field supervisors and allocated randomly to one of the intervention groups. Prior to enrolment, an information sheet was read to the caregiver for signing and informed consent was obtained. After six months of supplementation and follow up, the children were assessed by the ASQ-3. There were no additional exclusion criteria for the ASQ-3 assessment. Written and verbal consent was gathered from the caregiver separately for the ASQ-3 component of the study. The Data collection period lasted from May to September 2011.

### Instrument

ASQ-3 is a comprehensive checklist of developmental status, standardized for children 1-66 months with age-appropriate questionnaires. For earlier ages there are questionnaires for every two month interval, the intervals increase with age. The ASQ-3 has five subscales: Communication, Gross Motor, Fine Motor, Problem-Solving and Personal-Social. Each form contains 30 items, six for each subscale, written in a simple language. Some questions are specific for certain age groups, while other items are used for a wider age range and are repeated in the different age-specific questionnaires. The questionnaires are designed to be completed by caregivers, but can also be administered by a professional in the “home procedure” described in the ASQ-3 manual [9]. In the “home-procedure”, the professional plays an active part in the assessment of the child providing necessary material for the direct assessment of skills during the sessions. The “home procedure” is not necessarily performed in the home of the family, but in any given arena where the child, caregiver and professional meet.

### Procedure

#### Training

For 11 days immediately before initiation of the 5 months ASQ-data collection, three field supervisors (the examiners) were trained by the main author to perform the ASQ-3 “home procedure”. The main author is a clinical psychologist with experience in giving training in the assessment of infants and young children in similar projects. The field supervisors had experience in working in the local community with the study population, two had degrees at master’s level. None of the supervisors had formal training in developmental psychology. They were under the supervision of medical doctors, and responsible for the work of the field workers in the main study. During the 11 days training, administration, the understanding of the inherent ideas of items and scoring, as well as approaches and techniques in terms of rapport building was discussed and practiced. Standardization exercises were performed in 30 children aged 12-36 months during the same training.

#### Translation

Eleven ASQ-3 forms, covering the ages 12-36 months, were formally translated to Hindi, the spoken language in the area. The translation process followed recommended procedures [4]. The field supervisors were responsible for the forward translations. The translations were thereafter back-translated to English by an employee of the Society for Applied Studies, otherwise not involved in the study. The original forms and the back translations were then reviewed and discussed by the team. No adjustments were made to the initial translation following these discussions. The translations for each age group were typed and laminated, and then used as a support to the original ASQ-3 forms during sessions.

#### Cultural adjustments

The assessment room was decorated with culturally appropriate decorations. The assessments were performed on a carpet on the floor as suitable for the children in the community. Materials for the sessions were purchased at local markets, and were considered to be correct for the local community. For some communication items, pictures were downloaded from the Internet in order to ensure appropriateness for the culture, (i.e. child that eats and child that plays with a ball).

Each item in the 11 relevant forms was discussed in the local research team (physician and field supervisors) in order to adapt the ASQ-3 to the study population. The feasibility of each item, possible difficulties in the translation process and possible challenges in the transition to the local community were discussed. Some small necessary adjustments were discussed and made to various items. These include slight changes of the examples in items to ensure the cultural appropriateness. I.e. in a communication item, examples of directions for the child were slightly adjusted from: “*Find my coat”* to: “*Find my shoe”,* and from: *“Get your book”* to: *“Get your (other relevant belonging during the session)”.*

Four items were identified not to be appropriate for the study population and were changed. These were an item involving a fork, an item involving a zipper, an item involving a mirror and one requiring the child’s knowledge of both his/hers first and last name. Forks are not regular utensils in this area, and in the Personal Social subscale, the concept of fork was replaced with the concept of chapatti (flat bread), and the item was changed from: “*Does your child eat with a fork?”* to: “*Does your child take chapatti with Dal (lenses)?* Children’s clothing in this area does not normally include zippers, and thus children would be unfamiliar with the concept of zippers. The ASQ-3 item involving a zipper is an item in the communication subscale and the aim of the item is to assess the child’s understanding of the concepts *up* and *down*, not the inherent capacities of a zipper. The zipper was therefore replaced with a magnet that the child could move up and down on a magnetic board on the wall. The concept of *zipper* was literary changed with the concept of *magnet* in the relatively long item text. Mirrors are not common in the study area, and many children would be unfamiliar in interacting with their mirror image and could not be expected to offer a toy to its image the first time interacting with a mirror. The item involving a mirror was changed from: *While looking at herself in the mirror, does your child offer a toy to her own image?* to: *While looking at herself in the mirror, does your child smile and interact with the reflection?* And finally, family names are seldom used in this population, and thus children in the study area are only expected to be familiar with their first name. In the discussions, the team found it difficult to find a replacement that would be reasonable, and the item was thus only slightly changed to involve only the first name. The item: “*When you ask, “What is your name?” does your child say both her first and last name?”* was changed to: *“When you ask, “What is your name?” does your child say her first name?*

#### Administration of the ASQ-3

The ASQ-3 assessment was performed immediately following the end study procedures for the main study by one of the three examiners in a rotational order. Appointments with the families were made ahead, and a field worker would escort the child and caregiver to the clinic. Due to caregivers’ possible inexperience with filling out questionnaires and/or illiteracy, a revised version of the “home procedure” outlined in the ASQ-3 manual was used. In the home procedure, the examiners try to elicit the relevant skills in the child during sessions, and necessary materials are natural parts of the assessments. Caregivers serve as important contributors in supporting their child as well as in providing help to elicit behaviours. The approach allowed for the assessment of skills with materials that the children often would not possess in their homes, i.e. beads and blocks. During the assessments the examiners gave the child time to play and practice (approximately five minutes) with the relevant material, and scored the items based on the child’s accomplishment after the brief time of practice. The materials necessary for the screening were gathered in a standardized “Material kit”, ensuring that the same materials were used in every session. Assessment would last for approximately 20-30 minutes. Each item was scored according to the ASQ-3 manual, Yes, Sometimes, and Not Yet. The sessions started with “ice-breaking” sessions, where the children played with toys while the caregiver received relevant information for the ASQ-3 session from the caregiver and additional demographic information was gathered. Throughout the sessions the examiner held a friendly and non-threatening atmosphere in order to make both the child and caregiver comfortable in the new setting. When necessary, the examiners used toffees as motivators.

The examiners intention was to observe as much of the relevant skills and abilities as possible during the sessions, and not to rely solely on caregivers report on whether the child had developed the relevant skill. However, some items were not possible to assess during sessions, i.e. “*Does the child feed him/herself with a spoon”*, and the examiner would then have to rely on the mothers` information. For every item, the examiners noted whether the scoring was based on examiners observation or the caregiver’s report.

Following the completion of the ASQ-3 assessment, caregivers received a brief feedback of the child’s performance during the session, including small advice on relevant developmental topics when this was considered necessary by the examiner.

### Inter-observer agreement

Standardization exercises during the initial training, and the testing of ten % of sessions by two examiners during the main study were initiated to measure and ensure appropriate inter-observer agreement. The standardization exercises were performed in 30 children within the 11-day training period. The examiners alternated in performing the assessment, while the others observed and scored the session together with the first author who served as the gold standard. In addition, during the main study, ten % of the assessments, a total of 42 sessions, were randomly selected and scored by two examiners.

### Statistical analyzes

Double data entry by two data encoders followed by validation was completed within 48 hours of the assessment sessions. Data was coded according to the ASQ-3 manual, 0 = Not yet, 5 = Sometimes and 10 = Yes. For missing items, we followed the instructions from the ASQ-3 manual and an adjusted total score was computed by dividing the total subscale score by the number of items answered in that scale, and then adding this number depending on the amount of items missing [9]. We calculated the Intra-class correlation coefficient (ICC), when comparing any of the examiners with the gold standard (first author) during the exercises, and when comparing two examiners during the data collection. Pearson product moment correlation coefficient was calculated between the five subscales and the total ASQ-3 score. Standardized alpha values, as appropriate when standard scores are summed to form scale scores [18], were calculated for the total score and for the five subscales for the 11 age intervals. Alpha values greater than 0.80 were considered to indicate high internal consistency, values from 0.60 to 0.80 were considered satisfactory, and alpha values from 0.40 to 0.60 were considered moderately internally consistent. Data was analyzed in Stata (StataCorp, College Station, TX), version 12.

## Results

Of the 440 children included in the ASQ-3 assessment study, 18 children were not available at the scheduled visit; the total number of children assessed with the ASQ-3 was accordingly 422. All 422 available children completed their session. In the 422 completed questionnaires, 0.21% of the responses were missing.

### Background characteristics

Table 1 shows the demographic information of the children assessed with the ASQ-3. Two hundred and fifty-nine were younger than 23 months. There was an even distribution of girls and boys. At baseline, most of the children were breastfed (86.3%), ten % of the children were wasted, 40.1% were stunted, and 31.0% were underweight. Average years of schooling for the fathers and mothers were 8.6 and 7.0 years, respectively. Of the fathers, 99.1% worked, while only 6.1% of the mothers reported to have work outside of the home. A total of 194 children lived in a joint family and the family size ranged from 3-25 with an average of 5.8 family members. The average number of children in the families was three.

**Table 1:** Demographic characteristics of children in the cohort

	**N**	**Mean/%**	**SD**
**Child characteristics**			
Total	422		
Age in month			
12-23 months	259	61.3%	
24-36 months	163	38.7%	
Sex			
Girls	206	48.8%	
Boys	216	51.2%	
Breastfeeding status at baseline	364	86.3%	
Baseline Z score weight for length (wasted), < - 2, n (%)	42	10%	
Baseline Z score length for age (stunted), < -2, n (%)	169	40.1%	
Baseline Z score weight for age (underweight), < -2, n (%)	131	31%	
**Family situation**			
***Economy***			
Annual income (median/range in 1000 rupees)		73000	12000-870000
Families who own colour TV, scooter or cooler, n (%)	377	89.3%	
***Maternal characteristics***			
Age		25.7	5.5
Years of schooling		7	6.3
Mothers who work, n (%)	27	6.1%	
***Paternal characteristics***			
Years of schooling		8.6	4
Fathers who work, n (%)	418	99.1%	
***Household characteristics***			
Type of family			
Nuclear, n (%)	228	54%	
Joint, n (%)	194	46%	
Number of children in the family		3	2.3
Family size		5.8	2.6

### Inter-observer agreement

Table 2 shows the ICCs between the gold standard (first author) and examiner one, two and three in the standardization exercises during training. All values show strong correlations between the different examiners and the gold standard across subscales and on the total scores. Table 3 shows the ICCs of the quality checks between examiner one and two during the supplementation trial, all values are consistently strong.

**Table 2:** Intra class correlations between the gold standard and examiner 1, 2 and 3 during training exercises

	**N**	**Total score**	**Communication**	**Gross motor**	**Fine motor**	**Problem solving**	**Personal social**
		**ICC**	**ICC**	**ICC**	**ICC**	**ICC**	**ICC**
		**(95% CI)**	**(95% CI)**	**(95% CI)**	**(95% CI)**	**(95% CI)**	**(95% CI)**
**Examiner 1**	27	0.99	0.99	0.97	0.90	0.94	0.99
		(0.98-1)	(0.99-1)	(0.95-0.99)	(0.83-0.97)	(0.90-0.99)	(0.98-1)
**Examiner 2**	30	0.98	0.99	0.97	0.86	0.96	0.97
		(0.97-0.99)	(0.99-1)	(0.94-0.99)	(0.77-0.95)	(0.93-0.99)	(0.95-0.99)
**Examiner 3**	30	0.99	0.99	0.99	0.96	0.89	0.99
		(0.98-1)	(0.99-1)	(0.99-1)	(0.92-0.99)	(0.82-0.96)	(0.98-1)

**Table 3:** Intra class correlation between examiner 1 and examiner 2 during the main study

	**Total score**	**Communication**	**Gross motor**	**Fine motor**	**Problem solving**	**Personal social**
ICC	0.95	0.91	0.92	0.94	0.91	0.90
(95% CI)	(0.93-0.98)	(0.86-0.96)	(0.88-0.97)	(0.91-0.98)	(0.86-0.96)	(0.84-0.96)

### Source of information for the scoring of items

**Figure 1:**
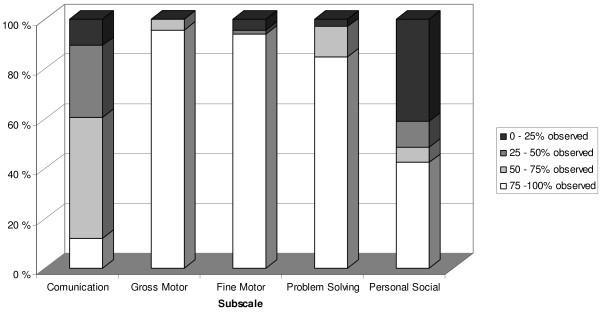
**Percentage of items in each subscales observed by examiners during sessions.** For different subscales there were variations on the number of items observed during sessions. The figure shows these differences for each subscale in percentages.

For different subscales, there were variations on the number of items observed during sessions and items scored based on caregivers report, see Figure 1 for details on the percentage of observed items during sessions. The Gross Motor, Fine Motor and the Problem Solving subscale were the subscales where most items were observed by the examiners. In the Communication and Personal Social subscales, more items are based on caregiver’s report of children’s relevant skills. The Personal Social is the subscale where the least of the items were based on the examiners observations during sessions.

### Variability

Means and standard deviation for the total score and the five subscales are summarized in Table 4. For the 422 assessed children, the mean total ASQ-3 score was 231.9 (SD = 50) with scores ranging from a minimum of 30 to a maximum of 300. For the subscales the mean scores range from 44.8 to 47.8, all with a range from zero to 60. However, across all age levels, 18 of the total 330 (5.4%) items administered showed no variations in the scoring (constant items), because all participants had developed the relevant skill for these items. Ten of these items were in the Gross motor subscale, five in the Personal Social subscale and one in each of the remaining subscales. Five of the constant items in the Gross motor subscale are assessing the child’s ability to walk and run on different age levels (i.e. Gross motor item 3, 18 months: *Does your child walk well and seldom fall?* and Gross motor item 4, 24 months: *Does your child run fairly well, stopping herself without bumping into things or falling?).* Two of the constant items in the Personal Social subscale are the same on different age categories and are assessing the ability to drink from a cup or glass (i.e. Personal Social item 4, 22 months: *Does your child drink from a cup or glass, putting it down again with little spilling?*). None of the constant items were items that were adjusted during the translation process.

**Table 4:** Means, standard deviation and range of the total ASQ-scale score and the five subscale scores for all children

	**N**	**Total scale**	**Communication**	**Gross motor**	**Fine motor**	**Problem solving**	**Personal social**
**Mean (SD)**	422	231.9 (50)	47.8 (15.4)	46.2 (14.1)	46.7 (13.5)	44.8 (13.9)	46.4 (12.4)
**Range**	422	30-300	0-60	0-60	0-60	0-60	0-60

### Internal consistency

The Pearson product moment correlation coefficients across all age levels between the total ASQ-3 score and the subscales and between the different subscales are shown in Table 5. The correlations between the total ASQ-3 scores and the subscales are strong and the correlations between the five subscales are moderate.

**Table 5:** Pearson product moment correlation coefficients between the five subscales across age levels and the total ASQ-score

	**Communication**	**Gross motor**	**Fine motor**	**Problem solving**	**Personal social**
**Communication**					
**Gross Motor**	0.37				
**Fine Motor**	0.37	0.36			
**Problem Solving**	0.50	0.35	0.52		
**Personal Social**	0.31	0.39	0.43	0.41	
**Total**	0.73	0.69	0.73	0.77	0.68

0.5-1 = strong, 0.3-0.5 = moderate and 0-0.3 = weak correlation.

All correlations are significant at p < .000.

Table 6 shows the standardized alphas for the total ASQ-3 score and the five subscales by age interval. The 66 alpha values span from negative values (no values in the table) to .92. In the Communication subscale, three values are within highly internal consistency and four values are within satisfactory. In the Gross Motor subscale, two values are highly internally consistent and three values are satisfactory. In Fine Motor and Problem Solving, six values are satisfactory. In Personal Social three values are satisfactory. In this last subscale the values for 24 and 36 months are marked as missing due to negative average covariance. For 24 months two items (item 4 and 5) were identified causing the negative average covariance. Item 4 is a question concerning the child’s pretend play with a stuffed animal or doll and item 5 is on the child`s ability to steer around objects and back out of corners when pushing a little wagon, stroller or other toy on wheels. For 36 months one item (item 6) was identified to cause negative average covariance, this item is on the child`s ability to take turns by waiting while another child or adult takes a turn.

**Table 6:** Standardized alphas by total ASQ-3 scale and subscales

**Age interval**	**N**	**Total scale**	**Communication**	**Gross motor**	**Fine motor**	**Problem solving**	**Personal social**
		**Std. α**	**Std. α**	**Std. α**	**Std. α**	**Std. α**	**Std. α**
**12**	51	0.77	0.33	0.69	0.62	0.62	0.53
**14**	43	0.88	0.46	0.82	0.54	0.78	0.57
**16**	37	0.90	0.67	0.90	0.76	0.69	0.67
**18**	34	0.75	0.58	0.47	0.62	0.46	0.26
**20**	42	0.90	0.81	0.73	0.66	0.71	0.76
**22**	34	0.72	0.70	0.16	0.49	0.17	0.43
**24**	39	0.82	0.76	0.58	0.29	0.49	-*
**27**	47	0.84	0.73	0.64	0.59	0.45	0.36
**30**	37	0.84	0.57	0.35	0.63	0.51	0.70
**33**	42	0.87	0.81	0.48	0.71	0.63	0.50
**36**	16	0.91	0.92	0.28	0.30	0.70	-*

α >0.80 - highly, α = 0.60-0.80 – satisfactory, and α = 0.40-0.60 – moderate internally consistent.

* negative average covariance.

To assess the effect of the four adapted items on the internal consistency, further analysis were performed in the relevant subscales and age groups. Removing the adapted item increased the alpha value of Personal Social, 27 month from 0.36 to 0.45, while in the communication subscale for 33 and 36 months, the values decreased from 0.81 to 0.64 and 0.92 to 0.87 respectively. For Personal Social 16, 18, 20 and 24 months the values remained unchanged.

## Discussion

The feasibility of the ASQ-3 “home procedure” was assessed in a field trial in a North Indian urban setting. Ahead of the five months data collection, there was an 11 days training, including translation, cultural adjustments and standardization of the examiners. In the translational process, four items were changed in order to be appropriate for the study population. The general feedback both during the training of the examiners and during the clinical trial, indicated that children, caregivers and examiners commonly found the ASQ-3 “home procedure” enjoyable to attend. The examiners experienced the ASQ-3 “home procedure” as a reasonable and feasible instrument to administer in the current clinical trial. During the study, all initiated sessions were completed and with very few missed observations. The ICC-values show a high degree of inter-observer agreement both during standardization and the main study, indicating feasibility of the ASQ-3 in terms of the collection of reliable data.

Furthermore, the total ASQ scores were in the entire range of possible values, however, some items did not show any variability. The correlation coefficients showed satisfactory concurrence between the five subscales and the total scale, but the standardized alpha values varied in the different subscales and age levels indicating some weakness of the internal consistency.

For the cultural adjustments of the ASQ-3 forms, four items in the 11 relevant forms were identified as improper in a North Indian context, and were changed. This is in accordance with other studies on the translation and adjustments of the ASQ to new cultural contexts reporting similar changes at item level [10,11,17]. It may seem that some items are more challenging to use in other cultures. For example, the item concerning a fork was changed in the present study, likewise a study in Ecuador reports that items involving using a fork were removed as they are not commonly used [17]. In the previously mentioned study from India, the items with forks were also mostly left unanswered indicating that the items were irrelevant for the children in the sample [14]. Furthermore, mirrors were found to be uncommon for the present study population, also demonstrated in previous adaptions [14]. In the ASQ-3 manual, the mirror-items are highlighted as possible problematic items for many cultures [9]. This could suggest that there are some items that are more cultural specific than others, and which should be considered with particular care while interpreting the results from studies, as well as when necessary adjustments are made in future studies.

In the present study eighteen items showed no variability since all children in the specific age categories had developed the relevant skill for the item, for example the skill of walking in the Gross Motor subscale. This might have been incidental since groups at each age levels were small (ranging from 16 to 52 participants in each age category). However, the number of constant items may also be an expression of cultural differences in child rearing practices and expectations to children’s development between North India and the US. This last assumption gives rise to the idea that the 18 constant items are not developmentally appropriate for this North Indian sample of infants and young children, and should be adjusted and/or regrouped age appropriately prior to further use.

The internal consistency of the ASQ-3 when transferred to a North Indian setting was expressed by correlations between the total scores and the subscale scores, and by standardized alphas. The strong and consistent correlation coefficients between all of the five subscales and the total ASQ-3 scale indicate concurrency. The moderate correlation coefficients between the five subscales are expected, indicating a certain degree of concurrence between the subscales, but at the same time underlining that the subscales measure different developmental skills. These results are in accordance with the correlations between the different subscales and the total scores described in the ASQ-3 manual [9]. For the standardized alphas however, the picture is not as clear. The 66 alpha values range from highly internally consistent to unsatisfactory and in two instances, negative values. The standardized alphas for the total scale at the different age groups generally indicate that the scale is highly internally consistent and measuring the same thematic areas. For the subscales however, the values vary. The calculations of the standardized alphas therefore unfold additional problematic items causing unsatisfactory alpha values, and even, negative item covariance. These items are inconsistent with the other items in the subscale, and therefore might not assess the same developmental area in this setting. Analysis on relevant subscales when removing adapted items does not consistently lead to improved internal consistency, and thus indicating that these are not the primary cause of the poor internal consistencies. The problematic items should be scrutinized further in order to get an understanding to why certain items in this cultural setting show inconsistency. With further adjustments to certain items there might be a possibility to improve the internal consistency of the scales, and then increase the level of reliability.

The calculations of the standardized alphas are sensitive to the number of items that are included in the analysis [18]. In the alpha calculations of the total scale, 30 items are included, while only six items are included in the calculations of the subscale alphas. Constant items are excluded from the analysis of standardized alphas, and therefore, the number of items may be even fewer than six on certain age levels in this study since a total of 18 items are constant. This may reduce the alpha values in the relevant subscales and age levels even further. Two alpha values are particular problematic in our calculations. These are in the Personal Social scale at 24 and 36 months where items cause negative average covariance, and therefore violate the assumptions of the calculations, resulting in no alpha values shown in the results.

In the technical report of the ASQ-3 manual, the standardized alpha values from their sample of 18 000 children are listed. It was concluded that the overall internal consistency of the subscales was good to acceptable. However, the table of the alphas for all the age intervals has values from 0.51 to 0.87. The Personal Social subscale is the scale with the poorest values. In a study on the cross cultural adaption of the ASQ-2 to a Korean setting, the standardized alpha values of all subscales ranged from 0.30 to 0.91, again with the poorest values in the Personal Social subscale [11]. In their discussion of the study, Heo, Squires and Yovanoff [11] argue that Personal Social items such as eating and dressing skills will give rise to differences between the Korean and the US sample. Gladestone et al. [19] argue similarly in their report on the modification of Western screening tools to a Malawian setting that cultural differences often appear in the area of social development. These assumptions are in accordance with the present study, where the Personal Social subscales offers the overall poorest alpha values. In the process of further adjusting the ASQ-3 to a North Indian setting, the Personal Social subscale should be handled with particular care.

We administered the ASQ-3 as “home procedure”. Feedback and observations during the sessions indicate that the ASQ-3 “home procedure” in general was an enjoyable time both for children and caregivers. Examiners experienced the adjusted ASQ-3 as reasonable in assessing children from the area. This indicates that the face validity of the adjusted ASQ-3 was satisfactory. Sessions were brief and all 422 children completed their session once it was initiated. Children were given time during sessions to practice with possible unfamiliar material and were scored based on their accomplishments during sessions. Based on the possibility of collecting information both from observation and caregiver’s report missing data were scarce. These factors support the feasibility of the ASQ-3 “home procedure” in large population-based studies. Furthermore, the developmental assessment was conducted at a low cost. The examiners were not psychologists, the ASQ-3 kit was purchased online, and only one kit was required for the study site. Necessary materials and equipment for the “home procedure” were purchased at local markets, or downloaded from the Internet. Accessible tools at low cost, that are easy to use and which are enjoyable for the children in a given culture are in accordance with the recommendations of Fernald, Kariger, Engle and Raikes [3] in their toolkit for the assessment of child development in low and middle-income countries.

However, the “home procedure” approach does require some training of examiners, in addition to practice sessions after the initial training. In our study we conducted an 11 days training, which also included discussions of cultural adjustments. The ICCs both of the standardization exercises during training and the quality check during the study period show that the examiners through intensive training and subsequent practice managed to obtain a high degree of concurrence in their scorings. The satisfactory ICCs serve as further support that the ASQ-3 “home procedure” may be a beneficial approach to efficiently obtain reliable data on child developmental status for research purpose.

A challenge of the ASQ-3 “home procedure” for research purpose is that, although examiners intention was to observe as much of the children’s skills during sessions as possible, some ASQ items fail to provide this possibility due to its inherent structure. Analysis shows that the Motor scales and the Problem Solving scales include most items that may be observed by examiners during an assessment session. The two remaining scales, Communication and Personal Social include more items that require information from the caregiver to score. The scales may therefore be perceived to provide data of different quality, three of the scales provide objective information scored by trained examiners, and two of the scales are more reliant of the subjective report from caregivers.

Parental report do provide a risk of inaccuracy and/or overstatements in the report of the child’s development due to factors such as social desirability, caregivers inexperience in interpreting their child’s skills and/or their inability to accurately report the child’s behaviour [3]. However, the ASQ-system is developed and based on the conviction that caregivers can provide information for proper assessments of their children. For instance, a study comparing the ASQ completion of low and middle-income parents in the US with subsequent assessment by the Bayley Scale of Infant and Toddler Development, shows no differences in the accuracy of scoring in the two groups of parents, giving support to the idea that parents-completion of child development questionnaires give reliable data also in high risk groups [20]. For now, when utilizing the ASQ-3 “home procedure” for research purpose in this cultural setting, data should be carefully interpreted with the difference in the quality of information in mind.

The total ASQ-3 scores range from zero (no scores) to 300 (full score), in our study the scores ranged from 30 to 300. The five subscales ranged from zero to 60 (full subscale score). Our results imply that although the data are not perfectly normally distributed, the ASQ-3 managed to identify children in both ends of the scale. The total ASQ-scores has a mean of 231.9 and SD of 50, while for the subscales the mean scores range from 44.8 to 47.8. A study by Kerstjens et al. [13] compares mean subscale values between Dutch, US, Norwegian and Korean samples. The mean values from our study are generally lower on all subscales, except for the Fine motor subscale were mean values from our studies are slightly larger than in the Dutch and US sample, but still lower than in the Norwegian and Korean sample. The intention of this study has not been to formally validate the ASQ-3 for a North Indian setting and establishing cut off scores for developmental delay in the children. The differences of mean subscale values should therefore be interpreted with care. Fernald, Kariger, Engle and Raikes [3] emphasize that when cut off scores are not established for the given culture were the screening tool is used, its use should be limited to that of comparing groups. The differences between mean values in our study from other studies underline this statement. Until further validation has been conducted on the ASQ-3 for this particular population, there are no cut-off scores feasible for this North Indian sample, and data should be limited to the comparison of groups.

When evaluating the transference of an assessment tool to a new cultural context, test-retest reliability is of importance. Within the framework of this study, such evaluation was not possible. This is a definite weakness of the study. Furthermore, piloting of the translated questionnaire prior to the study would be preferable, and give room for further adjustments ahead of the study start based on preliminary calculations of internal consistencies, variability and constant items. These limitations of the study, together with other remarks in the Discussion section should set the groundwork for further attempts to transfer the ASQ-3 to new cultural settings.

## Conclusion

The present study has evaluated the feasibility of the ASQ-3 “home procedure” as an easily administered and inexpensive assessment tool for the collection of data on developmental status in infants and young children in an epidemiological study in a North Indian urban setting. Our results are promising in terms of the possibility to effectively train examiners to collect reliable data in a large study. However, for future utility in similar research setting, particular attention must be held to further adjustments of items, as well as the possibility of re-grouping items more age-appropriately, in order to enhance the internal consistency of the scales. The report underlines the significance of close awareness to cultural adjustments when transferring an assessment tool to a new cultural context, both in terms of translation and adaptation of items and in terms of cultural appropriate administration.

## Competing interests

The authors declare that they have no competing interests.

## Authors’ contributions

NB, ST, TAS, TK, MH and IK designed research; TK, IK and Study group conducted research; NB, ST, TK and TAS were responsible for the data management; TAS and IK performed statistical analysis, MH and IK wrote the manuscript, MH, TAS and IK had the primary responsibility of the final content. All authors contributed to the writing and approved the final version of the manuscript.

## Authors’ information

Study group: Sanjana Mohan, Madhu Mahesh, Pooja Gupta, Divya Pandey, Pankaj Bhardwaj and Vandna Suri. Society for Essential Health Action and Training, New Delhi, India.

Lead author for study group: Sunita Taneja.
